# Confidence interval for micro-averaged *F*_1_ and macro-averaged *F*_1_ scores

**DOI:** 10.1007/s10489-021-02635-5

**Published:** 2021-07-31

**Authors:** Kanae Takahashi, Kouji Yamamoto, Aya Kuchiba, Tatsuki Koyama

**Affiliations:** 1 Department of Medical Statistics, Osaka City University Graduate School of Medicine, Osaka, Japan; 2 Department of Biostatistics, Hyogo College of Medicine, Hyogo, Japan; 3 Department of Biostatistics, School of Medicine, Yokohama City University, Yokohama, Japan; 4 Graduate School of Health Innovation, Kanagawa University of Human Services, Kanagawa, Japan; 5 Division of Biostatistical Research, Center for Public Health Sciences / Biostatistics Division, Center for Research Administration and Support, National Cancer Center, Tokyo, Japan; 6 Department of Biostatistics, Vanderbilt University Medical Center, Nashville, TN, USA

**Keywords:** Precision, Recall, Machine learning, *F*_1_ measures, Multi-class classification, Delta-method

## Abstract

A binary classification problem is common in medical field, and we often use sensitivity, specificity, accuracy, negative and positive predictive values as measures of performance of a binary predictor. In computer science, a classifier is usually evaluated with precision (positive predictive value) and recall (sensitivity). As a single summary measure of a classifier’s performance, *F*_1_ score, defined as the harmonic mean of precision and recall, is widely used in the context of information retrieval and information extraction evaluation since it possesses favorable characteristics, especially when the prevalence is low. Some statistical methods for inference have been developed for the *F*_1_ score in binary classification problems; however, they have not been extended to the problem of multi-class classification. There are three types of *F*_1_ scores, and statistical properties of these *F*_1_ scores have hardly ever been discussed. We propose methods based on the large sample multivariate central limit theorem for estimating *F*_1_ scores with confidence intervals.

## 1 Introduction

In medical field, a binary classification problem is common, and we often use sensitivity, specificity, accuracy, negative and positive predictive values as measures of performance of a binary predictor. In computer science, a classifier is usually evaluated with precision and recall, which are equal to positive predictive value and sensitivity, respectively. For measuring the performance of text classification in the field of information retrieval and of a classifier in machine learning, the *F* score (*F* measure) has been widely used. In particular, the *F*_1_ score has been popular, which is defined as the harmonic mean of precision and recall [1, 2]. The *F*_1_ score is rarely used in diagnostic studies in medicine despite its favorable characteristics. As a single performance measure, the *F*_1_ score may be preferred to specificity and accuracy, which may be artificially high even for a poor classifier with a high false negative probability when disease prevalence is low. The *F*_1_ score is especially useful when identification of true negatives is relatively unimportant because the true negative rate is not included in the computation of either precision or recall.

To evaluate a multi-class classification, a single summary measure is often sought. And as extensions of the *F*_1_ score for the binary classification, there exist two types of such measures: a micro-averaged *F*_1_ score and a macro-averaged *F*_1_ score [2]. The micro-averaged *F*_1_ score pools per-sample classifications across classes, and then calculates the overall *F*_1_ score. Contrarily, the macro-averaged *F*_1_ score computes a simple average of the *F*_1_ scores over classes. Sokolova and Lapalme [3] gave an alternative definition of the macro-averaged *F*_1_ score as the harmonic mean of the simple averages of the precision and recall over classes. Both micro-averaged and macro-averaged *F*_1_ scores have a simple interpretation as an average of precision and recall, with different ways of computing averages. Moreover, as will be shown in Section 2, the micro-averaged *F*_1_ score has an additional interpretation as the total probability of true positive classifications.

For binary classification, some statistical methods for inference have been proposed for the *F*_1_ scores (e.g., [4]); however, the methodology has not been extended to the multi-class *F*_1_ scores. To our knowledge, methods for computing variance estimates of the micro-averaged *F*_1_ score and macro-averaged *F*_1_ score have not been reported. Thus, computing confidence intervals for the multi-class *F*_1_ scores is not possible, and the inference about them is usually solely based on point estimates, and thus highly limited in practical utility. For example, consider the results of an analysis reported by Dong et al. [5]. In this analysis, the authors calculated the point estimates of macro-averaged *F*_1_ scores for four classifiers, and they concluded a classifier outperformed the others by comparing the point estimates without taking into account their uncertainty. Others have also used multi-class *F*_1_ scores but only reported point estimates without confidence intervals [6–16].

To address this knowledge gap, we provide herein the methods for computing variances of these multi-class *F*_1_ scores so that estimating the micro-averaged *F*_1_ score and macro-averaged *F*_1_ score with confidence intervals becomes possible in multi-class classification.

The rest of the manuscript is organized as follows: The definitions of the micro-averaged *F*_1_ score and macro-averaged *F*_1_ score are reviewed in Section 2. In Section 3, variance estimates and confidence intervals for the multi-class *F*_1_ scores are derived. A simulation study to investigate the coverage probabilities of the proposed confidence intervals is presented in Section 4. Then, our method is applied to a real study as an example in Section 5 followed by a brief discussion in Section 6.

## 2 Averaged *F*_1_ scores

This section introduces notations and definitions of multi-class *F*_1_ scores, namely, macro-averaged and micro-averaged *F*_1_ scores. Consider an *r* × *r* contingency table for a nominal categorical variable with *r* classes (*r* ≥ 2). The columns indicate the true conditions, and rows indicate the predicted conditions. It is called the binary classification when *r* = 2, and the multi-class classification when *r* > 2. Such a table is also called a confusion matrix. We consider multi-class classification, i.e., *r* > 2, and denote cell probabilities and marginal probabilities by *p*_*ij*_, *p*_*i*·_, and *p*_·*j*_, respectively (*i*, *j* = 1, ⋯, *r*). For each class (*i* = 1, ⋯, *r*), the true positive rate (*TP*_*i*_), the false positive rate (*FP*_*i*_), and the false negative rate (*FN*_*i*_) are defined as follows:

$$TP_{i}=p_{ii},$$

$$FP_{i}=\sum_{\begin{array}{c} j=1\\ j\neq i \end{array}}^{r}p_{ij},$$

$$FN_{i}=\sum_{\begin{array}{c} j=1\\ j\neq i \end{array}}^{r}p_{ji}.$$

*TP*_*i*_ is the *i*-th diagonal element, *FP*_*i*_ is the sum of off-diagonal *i*-th row, and *FN*_*i*_ is the sum of off-diagonal elements of the *i*-th column. Note that *TP*_*i*_ + *FP*_*i*_ = *p*_*i*·_, and *TP*_*i*_ + *FN*_*i*_ = *p*_·*i*_.

In the current and following sections, we will use the simple 3-by-3 confusion matrix in Table 1 as an example to demonstrate various computations. Columns represent the true state, and rows represent the predicted classification. The total sample size is 100.

The within-class probabilities are:

$$TP_{1}=0.02,\;TP_{2}=0.70,\;TP_{3}=0.15.$$

$$FP_{1}=0.04,\;FP_{2}=0.07,\;FP_{3}=0.02.$$

$$FN_{1}=0.05,\;FN_{2}=0.04,\;FN_{3}=0.04.$$

### Micro-averaged *F*_1_ score

The micro-averaged precision (*miP*) and micro-averaged recall (*miR*) are defined as

$$miP=\frac{\sum_{i=1}^{r}TP_{i}}{\sum_{i=1}^{r}\left(TP_{i}+FP_{i}\right)}=\frac{\sum p_{ii}}{\sum p_{i\cdot }}=\sum_{i=1}^{r}p_{ii},$$

$$miR=\frac{\sum_{i=1}^{r}TP_{i}}{\sum_{i=1}^{r}\left(TP_{i}+FP_{i}\right)}=\frac{\sum p_{ii}}{\sum p_{\cdot i}}=\sum_{i=1}^{r}p_{ii}.$$

Note that for both *miP* and *miR*, the denominator is the sum of all the elements (diagonal and off-diagonal) of the confusion matrix, and it is 1. Finally, the micro-averaged *F*_1_ score is defined as the harmonic mean of these quantities:

(1) $$miF_{1}=2\frac{miP\times miR}{miP+miR}=\sum_{i=1}^{r}p_{ii}.$$

This definition is commonly used (e.g., [6, 8–12, 14, 15]).

By definition, we have *miP*, *miR*, and *miF*_1_ all equal to the sum of the diagonal elements, which, in our example, is 0.87.

### Macro-averaged *F*_1_ score

To define the macro-averaged *F*_1_ score (*maF*_1_), first consider the following precision (*P*_*i*_) and recall (*R*_*i*_) within each class, *i* = 1, ⋯, *r*:

$$P_{i}=\frac{TP_{i}}{\left(TP_{i}+FP_{i}\right)}=p_{ii}/p_{i\cdot },$$

$$R_{i}=\frac{TP_{i}}{\left(TP_{i}+FN_{i}\right)}=p_{ii}/p_{\cdot i}.$$

For our example, simple calculation shows:

$$P_{1}=0.33,\;P_{2}=0.91,\;P_{3}=0.88,$$

$$R_{1}=0.29,\;R_{2}=0.95,\;R_{3}=0.79.$$

And *F*_1_ score within each class (*F*_1*i*_) is defined as the harmonic mean of *P*_*i*_ and *R*_*i*_, that is,

$$F_{1i}=2\frac{P_{i}\times R_{i}}{P_{i}+R_{i}}=2\frac{p_{ii}}{p_{i\cdot }+p_{\cdot i}}.$$

The macro-averaged *F*_1_ score is defined as the simple arithmetic mean of *F*_1*i*_:

(2) $$maF_{1}=\frac{1}{r}\sum_{i=1}^{r}F_{1i}=\frac{2}{r}\sum_{i=1}^{r}\frac{p_{ii}}{p_{i\cdot }+p_{\cdot i}}.$$

This score, like *miF*_1_ is frequently reported (e.g., [5–10, 13]).

*F*_1*i*_ and *maF*_1_ in our example are:

$$F_{11}=0.308,\;F_{12}=0.927,\;F_{13}=0.833.$$

$$maF_{1}=(0.308+0.927+0.833)/3=0.689.$$

### Alternative definition of Macro-averaged *F*_1_ score

Sokolova and Lapalme [3] gave an alternative definition of the macro-averaged *F*_1_ score ($maF_{1}^{*}$). First, macro-averaged precision (*maP*) and macro-averaged recall (*maR*) are defined as simple arithmetic means of the within-class precision and within-class recall, respectively.

$$maP=\frac{1}{r}\sum_{i=1}^{r}\frac{TP_{i}}{TP_{i}+FP_{i}}=\frac{1}{r}\sum_{i=1}^{r}\frac{p_{ii}}{p_{i\cdot }},$$

$$maR=\frac{1}{r}\sum_{i=1}^{r}\frac{TP_{i}}{TP_{i}+FN_{i}}=\frac{1}{r}\sum_{i=1}^{r}\frac{p_{ii}}{p_{\cdot i}}.$$

And $maF_{1}^{*}$ is is defined as the harmonic mean of these quantities.


(3) $$maF_{1}^{*}=2\frac{maP\times maR}{maP+maR}.$$

This version of macro-averaged *F*_1_ score is less frequently used (e.g., [11, 12, 16]). For our example,

maP=(0.02/0.06+0.70/0.77+0.15/0.17)/3=0.708.

maR=(0.02/0.07+0.70/0.74+0.15/0.19)/3=0.674.

$$maF_{1}^{*}=0.691.$$

In this example, the micro-averaged *F*_1_ score is higher than the macro-averaged *F*_1_ scores because both within-class precision and recall are much lower for the first class compared to the other two. Micro-averaging puts only a small weight on the first column because the sample size there is relatively small. This numeric example shows a shortcoming of summarizing a performance of a multi-class classification with a single number when within-class precision and recall vary substantially. However, aggregate measures such as the micro-averaged and macro-averaged *F*_1_ scores are useful in quantifying the performance of a classifier as a whole.

## 3 Variance estimate and confidence interval

In this section, we derive the confidence interval for *miF*_1_, *maF*_1_, and $maF_{1}^{*}$. We assume that the observed frequencies, *n*_*ij*_, for 1 ≤ *i* ≤ *r*, 1 ≤ *j* ≤ *r*, have a multinomial distribution with sample size *n* and probabilities

$$p={\left(p_{11},\cdots ,p_{1r},p_{21},\cdots ,p_{2r},\cdots ,p_{r1},\cdots ,p_{rr}\right)}^{T},$$

where “*T*” represents the transpose, that is

$$\left(n_{11},n_{12},\cdots ,n_{rr}\right)\sim \text{Multinomial}(n;p).$$

The expectation, variance, and covariance for *i*, *j* = 1, ⋯, *r*, are:

$$E\left(n_{ij}\right)=np_{ij},$$

$$\text{Var}\left(n_{ij}\right)=np_{ij}\left(1-p_{ij}\right),$$

$$\text{Cov}\left(n_{ij},n_{kl}\right)=-np_{ij}p_{kl},\;\text{for}\;i\neq k\;\text{or}\;j\neq l,$$

respectively, where $n=\sum_{i,j}n_{ij}$ is the overall sample size. The maximum likelihood estimate (MLE) of *p*_*ij*_ is ${\hat{p}}_{ij}=n_{ij}/n$. Using the multivariate central limit theorem, we have

$$\sqrt{n}(\hat{p}-p)\dot{\sim }\text{Normal}\left(0_{r^{2}},\text{diag}(p)-pp^{T}\right),$$

where $0_{r^{2}}$ is *r*^2^ × 1 vector whose elements are all 0, *diag*(***p***) is an *r*^2^ × *r*^2^ diagonal matrix whose diagonal elements are ***p***, and “⩪”represents “approximately distributed as.”

By invariance property of MLE’s, the maximum likelihood estimates of *miF*_1_, *maF*_1_, and $maF_{1}^{*}$, and other quantities in the previous section can be obtained by substituting *p*_*ij*_ by ${\hat{p}}_{ij}$. In the following subsections, we use the multivariate delta-method to derive large-sample distributions of $\hat{miF_{1}}$, $\hat{maF_{1}}$, and $\hat{maF_{1}^{*}}$.

### 3.1 **Confidence interval for** *miF*_1_

As shown in (1), $miF_{1}=\sum p_{ii}$, and the maximum likelihood estimate (MLE) of *miF*_1_ is

$$\hat{miF_{1}}=\sum_{i=1}^{r}{\hat{p}}_{ii}.$$

Using the multivariate delta-method (Appendix A), we have

$$\hat{miF_{1}}\dot{\sim }\text{Normal}\left(miF_{1},\text{Var}\left(\hat{miF_{1}}\right)\right),$$

where variance of $\hat{miF_{1}}$ is

(4) $$\text{Var}\left(\hat{miF_{1}}\right)=\left(\sum_{i=1}^{r}p_{ii}\right)\left(1-\sum_{i=1}^{r}p_{ii}\right)/n.$$

And a (1 – *α*) × 100% confidence interval of *maF*_1_ is

$$\hat{miF_{1}}\pm Z_{1-\alpha /2}\times \sqrt{\hat{Var}\left(\hat{miF_{1}}\right)},$$

where $\hat{Var}\left(\hat{miF_{1}}\right)$ is $\text{Var}\left(\hat{miF_{1}}\right)$ with {*p*_*ii*_} replaced by $\left\{{\hat{p}}_{ii}\right\}$, and *Z*_*p*_ denote the 100 *p*-th percentile of the standard normal distribution. Computation of $\hat{Var}\left(\hat{miF_{1}}\right)$ for our numeric example is straightforward using (4):

$$\hat{Var}\left(\hat{miF_{1}}\right)=(0.02+0.70+0.15)\times \{1-(0.02+0.70+0.15)\}/100=0.0336^{2}.$$

And a 95% confidence interval for *miF*_1_ is

0.87±1.960×0.0336=(0.804,0.936).

### 3.2 Confidence interval for *maF*_1_

The MLE of *maF*_1_ can be obtained by substituting *p*_*ii*_, *p*_·*i*_ and *p*_·*i*_ by their MLE’s in (2).

$${\hat{maF}}_{1}=\frac{2}{r}\sum_{i=1}^{r}\frac{{\hat{p}}_{ii}}{{\hat{p}}_{i\cdot }+{\hat{p}}_{\cdot i}}.$$

Again by the multivariate delta-method (Appendix B), we have the variance of $\hat{maF_{1}}$ as

$$\text{Var}\left(\hat{maF_{1}}\right)=\frac{2}{r^{2}}\left\{\sum_{i=1}^{r}\frac{F_{1i}\left(p_{i\cdot }+p_{\cdot i}-2p_{ii}\right)}{{\left(p_{i\cdot }+p_{\cdot i}\right)}^{2}}\left(\frac{p_{i\cdot }+p_{\cdot i}-2p_{ii}}{p_{i\cdot }+p_{\cdot i}}+\frac{F_{1i}}{2}\right)+\sum_{i=1}^{r}\sum_{j\neq i}\frac{p_{ij}F_{1i}F_{1j}}{\left(p_{i\cdot }+p_{\cdot i}\right)\left(p_{j\cdot }+p_{\cdot j}\right)}\right\}/n.$$

And a (1 – *α*) × 100% confidence interval of *miF*_1_ is

$$\hat{maF_{1}}\pm Z_{1-\alpha /2}\times \sqrt{\hat{Var}\left(\hat{maF_{1}}\right)},$$

where $\hat{Var}\left(\hat{maF_{1}}\right)$ is $\text{Var}\left(\hat{maF_{1}}\right)$ with {*p*_*ii*_} replaced by $\left\{{\hat{p}}_{ij}\right\}$. This computation is complex even for a small 3 by 3 table; an R code (Appendix D) was used to compute the variance estimate and a 95% confidence interval of *maF*_1_.


$$\hat{Var}\left(\hat{maF_{1}}\right)=0.0650^{2},0.69\pm 1.960\times 0.0650=(0.562,0.817).$$

### 3.3 Confidence interval form $maF_{1}^{*}$

To obtain the MLE’s of $maF_{1}^{*}$ we first substitute *p*_*ii*_, *p*_*i*_ and *p*_·*i*_ by their MLE’s of *maP* and *maR* and use these in (3):

$$\hat{maF_{1}^{*}}=2\frac{\hat{maP}\times \hat{maR}}{\hat{maP}+\hat{maR}}.$$

As shown in Appendix C,

$$\text{Var}\left(\hat{maF_{1}^{*}}\right)=4n\frac{maR^{4}Var(\hat{maP})+2maP^{2}maR^{2}\text{Cov}(\hat{maP},\hat{maR})+maP^{4}Var(\hat{maR})}{{\left(maP+maR\right)}^{4}}/n,$$

where

$$Var(\hat{maP})=\frac{1}{r^{2}}\left(\sum_{i=1}^{r}\frac{p_{ii}\left(\sum_{j\neq i}p_{ij}\right)}{p_{i\cdot }^{3}}\right)/n,$$

$$Var(\hat{maR})=\frac{1}{r^{2}}\left(\sum_{i=1}^{r}\frac{p_{ii}\left(\sum_{j\neq i}p_{ji}\right)}{p_{\cdot i}^{3}}\right)/n,$$

$$\text{Cov}(\hat{maP},\hat{maR})=\frac{1}{r^{2}}\left(\sum_{i=1}^{r}\frac{\left(\sum_{j\neq i}p_{ij}\right)p_{ii}\left(\sum_{j\neq i}p_{ji}\right)}{p_{i\cdot }^{2}p_{\cdot i}^{2}}+\sum_{i=1}^{r}\sum_{j\neq i}\frac{p_{ii}p_{ij}p_{jj}}{p_{i\cdot }^{2}p_{j\cdot }^{2}}\right)/n.$$

A (1 – *α*) × 100% confidence interval of $maF_{1}^{*}$ is

$$\hat{maF_{1}^{*}}\pm Z_{1-\alpha /2}\times \sqrt{\hat{Var}\left(\hat{maF_{1}^{*}}\right)}.$$

Again to get $\hat{Var}\left(\hat{maF_{1}^{*}}\right)$ all components of $\hat{Var}\left(\hat{maF_{1}^{*}}\right)$ are replaced by their respective MLE’s. Using the accompanying R code (Appendix D), we computed the variance estimate and a 95% confidence interval of $maF_{1}^{*}$:

$$\hat{Var}\left(\hat{maF_{1}^{*}}\right)=0.0649^{2}\;0.69\pm 1.960\times 0.0649=(0.563,0.818).$$

## 4 Simulation

We performed a simulation study to assess the coverage probability of the confidence intervals proposed in Section 3. We set *r* = 3 (class 1, 2, 3), and generated data according to the multinomial distributions with ***p*** summarized in Table 2. The total sample size, *n*, was set to 25, 50, 100, 500, 1,000, and 5,000. For each combination of the true distribution and sample size, we generated 1,000,000 data, each time computing 95% confidence intervals for *miF*_1_, *maF*_1_, and $maF_{1}^{*}$.

In scenario 1, the true conditions of class 1, 2, and 3 have the same probability (1/3), and the recall and precision are equal (80%). Thus *miP* = *maP* = 0.80, *miR* = *maR* = 0.80, and $miF_{1}=maF_{1}=maF_{1}^{*}=0.80$.

In scenario 2, the true condition of class 1 has higher probability than the others (80% vs 10%), and the recall and precision of class 1 are also higher than the others (80% vs 40%, and 91% vs 27%, respectively). *miF*_1_ gives equal weight to each per-sample classification decision, whereas *miF*_1_ gives equal weight to each class. Thus, large classes dominate small classes in computing *miF*_1_ [2], and *miF*_1_ is larger than *maF*_1_ (*miF*_1_ = 0.72, *maF*_1_= 0.50, $maF_{1}^{*}=0.51$) in scenario 2 because class 1 has higher probability and has higher precision and recall.

In scenario 3, the true condition of class 1 has higher probability than the others (80% vs 10%). The precision of class 1 is higher than the others (94% vs 24%), and the recall of class 1 is lower than the others, (40% vs 80%). Compared to the other two scenarios, the diagonal entries are relatively small, which makes *miF*_1_ small (*miF*_1_ = 0.48, *maF*_1_ = 0.44, and $maF_{1}^{*}=0.55$).

Table 3 shows the coverage probability of the proposed 95% confidence intervals for each scenario. The coverage probabilities for both *miF*_1_ and *maF*_1_ are close to the nominal 95% when the sample size is large. When *n* smaller than 95%, especially for *maF*_1_ and $maF_{1}^{*}$. Moreover, computing a confidence interval for $maF_{1}^{*}$ for small *n* is often impossible because $\hat{maF_{1}^{*}}$ is undefined when either *p*_*i*·_ = 0 or *p*_·*j*_ = 0 for any *i* or *j*. In typical applications where these *F* scores are computed, *n* is large, and the small *n* problem is unlikely to occur.

## 5 Example

As an example, we applied our method to the temporal sleep stage classification data provided by Dong et al. [5]. They proposed a new approach based on a Mixed Neural Network (MNN) to classify sleep into five stages with one awake stage (W), three sleep stages (N1, N2, N3), and one rapid eye movement stage (REM). In addition to the MNN, they evaluated the following three classifiers: Support Vector Machine (SVM), Random Forest (RF), and Multilayer Perceptron (MLP). The data came from 62 healthy subjects, and classification by a single sleep expert was used as the gold standard. The staging is based on a 30-second window of the physiological signals called an EEG (electroencephalography) epoch. Thus, each subject contributes a large number of data to be classified. The total number of epochs depends on the classifiers, and it is about 59,000. Performance of each classifier was evaluated using *maF*_1_ along with precision, recall, and overall accuracy. They concluded that the MNN outperformed the competitors by comparing the point estimates of *maF*_1_ and overall accuracy. We provide here 95% confidence intervals for *miF*_1,_ *maF*_1_, and $maF_{1}^{*}$ for each of the four methods, as summarized in Table 4. The confidence intervals of *miF*_1_, *maF*_1_, and $maF_{1}^{*}$ for the MNN do not overlap with the point estimates of other methods, providing further evidence that MNN is superior to the other method. For completeness we present 95% confidence intervals for other methods in Table 4 as well. As *n* is large for this example, the confidence intervals are narrow, and the ones for MNN do not overlap with confidence intervals for other three methods.

## 6 Discussion

We derived large sample variance estimates of *miF*_1_, *maF*_1_, and $maF_{1}^{*}$ in terms of the observed cell probabilities and sample size. This enabled us to derive large sample confidence intervals.

Coverage probabilities of the proposed confidence intervals were assessed through the simulation study. According to the result of the simulation, when *n* is larger than 100, the coverage probability was close to the nominal level; however, for *n* < 100, the coverage probabilities tended to be smaller than the target. Moreover, with an extremely small sample size, $maF_{1}^{*}$ could not be estimated as computation of $maF_{1}^{*}$ requires all margins to be non-zero. Zhang et al. [17] have considered interval estimation *miF*_1_ and *maF*_1_ and proposed the highest density interval through Bayesian framework. On the other hand, we have proposed confidence interval for *miF*_1_, *maF*_1_, and *maF*_1*_ through frequentist framework using a large-sample approximation.

There is an inherit drawback of multi-class *F*_1_ scores that these scores do not summarize the data appropriately when a large variability exists between classes. This was demonstrated in the numeric example in Section 2 for which the within-class *F*_1_ values are 0.308, 0.927, and 0.833, and *miF*_1_, *maF*_1_, and $maF_{1}^{*}$ are 0.870, 0.689, and 0.691, respectively. Reporting multiple within-class *F*_1_ scores may be an option as done in [18] and [19]; however, an aggregate measure is useful in evaluating an overall performance of a classifier across classes. Another limitation with *F*_1_ scores is that they do not take into consideration the true negative rate, and they may not be an appropriate measure when true negatives are important.

For future works, we are working on developing hypothesis testing procedure for *miF*_1_, *maF*_1_, and $maF_{1}^{*}$ based on the variance estimates proposed in this article.

An R code for computing confidence intervals for *miF*_1_, *maF*_1_, and $maF_{1}^{*}$, available and presented in Appendix D.

## Figures and Tables

**Table 1:** Numeric example

		True Classification			
		Class 1	Class 2	Class 3	
a: Frequencies					
	Class 1	2	2	2	
Prediction	Class 2	5	70	2	
	Class 3	0	2	15	
b: Proportions					
	Class 1	0.02	0.02	0.02	0.06
Prediction	Class 2	0.05	0.70	0.02	0.77
	Class 3	0.00	0.02	0.15	0.17
		0.07	0.74	0.19	

**Table 2:** Simulation study: True cell probabilities

		True condition		
		1	2	3
Scenario 1				
	1	8/30	1/30	1/30
Predicted condition	2	1/30	8/30	1/30
	3	1/30	1/30	8/30
Scenario 2				
	1	64/100	3/100	3/100
Predicted condition	2	8/100	4/100	3/100
	3	8/100	3/100	4/100
Scenario 3				
	1	32/100	1/100	1/100
Predicted condition	2	24/100	8/100	1/100
	3	24/100	1/100	8/100

**Table 3:** Simulation study: Coverage probability

	Scenario 1			Scenario 2			Scenario 3		
*n*	*miF*_1_	*maF*_1_	$maF_{1}^{*}$	*miF*_1_	*maF*_1_	$maF_{1}^{*}$	*miF*_1_	*maF*_1_	$maF_{1}^{*}$
25	0.885	0.901	0.890	0.921	0.790	0.774	0.930	0.870	0.821
50	0.937	0.935	0.923	0.941	0.864	0.853	0.935	0.918	0.905
100	0.933	0.938	0.936	0.937	0.914	0.914	0.943	0.936	0.933
500	0.949	0.949	0.948	0.947	0.944	0.945	0.946	0.947	0.947
1000	0.946	0.948	0.948	0.947	0.947	0.947	0.947	0.949	0.947
5000	0.950	0.950	0.950	0.951	0.949	0.949	0.951	0.950	0.950

**Table 4:** Point estimates and confidence intervals for *miF*_1_, *maF*_1_, and $maF_{1}^{*}$

Method	*n*	$\hat{miF_{1}}$	95% CI	$\hat{maF_{1}}$	95% CI	$\hat{maF_{1}^{*}}$	95% CI
MNN	59,066	0.859	(0.856, 0.862)	0.805	(0.801, 0.809)	0.807	(0.803, 0.811)
SVM	59,255	0.797	(0.794, 0.800)	0.750	(0.746, 0.754)	0.756	(0.752, 0.760)
RF	59,193	0.817	(0.814, 0.820)	0.724	(0.720, 0.729)	0.746	(0.741, 0.750)
MLP	59,130	0.814	(0.811, 0.817)	0.772	(0.768, 0.776)	0.778	(0.774, 0.782)

## Appendix A: Derivation of the distribution and variance of $\hat{miF_{1}}$

Let ***p*** be the ordered elements of a confusion matrix. ***p*** = (*p*_11_, ⋯, *p*_1*r*_, *p*_21_, ⋯, *p*_2*r*_, ⋯, *p*_*r*1_, ⋯, *p_rr_*)^*T*^. Using the multivariate delta-method for $\hat{p}$, we get

(5)
$$\sqrt{n}\left(\hat{miF_{1}}-miF_{1}\right)\dot{\sim }\text{Normal}\left(0,{\left[\frac{\partial \left(miF_{1}\right)}{\partial (p)}\right]}^{T}\left(\text{diag}(p)-pp^{T}\right)\left[\frac{\partial \left(miF_{1}\right)}{\partial (p)}\right]\right).$$

Because $miF_{1}=\sum_{i=1}^{r}p_{ii}$ we have

$$\frac{\partial \left(miF_{1}\right)}{\partial \left(p_{ii}\right)}=1\;\forall i=1,\cdots r\;\text{and}\;\frac{\partial \left(miF_{1}\right)}{\partial \left(p_{ij}\right)}=0\;\text{if}i\neq j.$$

And

$$\frac{\partial \left(miF_{1}\right)}{\partial (p)}={\left(1,0,\cdots ,0,0,1,0,\cdots ,0,\cdots ,0,\cdots ,0,1\right)}^{T}.$$

Note that all the elements corresponding to the diagonal entries (*p*_*ii*_) of the confusion matrix is 1. To evaluate the variance in (5), further note that

$$\text{diag}(p)=\left(\begin{array}{ccccc} p_{11} & 0 & 0 & \cdots & 0\\ 0 & p_{12} & 0 & \cdots & 0\\ 0 & 0 & p_{13} & \cdots & 0\\ \vdots & & & \ddots & \\ 0 & 0 & 0 & \cdots & p_{rr} \end{array}\right),$$

$$pp^{T}=\left(\begin{array}{ccccc} p_{11}^{2} & p_{11}p_{12} & p_{11}p_{13} & \cdots & p_{11}p_{rr}\\ p_{12}p_{11} & p_{12}^{2} & p_{12}p_{13} & \cdots & p_{12}p_{rr}\\ p_{13}p_{11} & p_{13}p_{12} & p_{13}^{2} & \cdots & p_{13}p_{rr}\\ \vdots & & & \ddots & \\ p_{rr}p_{11} & p_{rr}p_{12} & p_{rr}p_{13} & \cdots & p_{rr}^{2} \end{array}\right).$$

Then we have

$${\left[\frac{\partial \left(miF_{1}\right)}{\partial (p)}\right]}^{T}(\text{diag}(p))\left[\frac{\partial \left(miF_{1}\right)}{\partial (p)}\right]=\left(p_{11},0,\cdots ,p_{22},0,\cdots ,p_{33},\cdots ,p_{rr}\right)\left[\frac{\partial \left(miF_{1}\right)}{\partial (p)}\right]=\sum_{i=1}^{r}p_{ii}{\left[\frac{\partial \left(miF_{1}\right)}{\partial (p)}\right]}^{T}\left(pp^{T}\right)\left[\frac{\partial \left(miF_{1}\right)}{\partial (p)}\right]=\left(\sum_{i=1}^{r}p_{ii}p_{11},\sum_{i=1}^{r}p_{ii}p_{12},\cdots ,\sum_{i=1}^{r}p_{ii}p_{rr}\right)\left[\frac{\partial \left(miF_{1}\right)}{\partial (p)}\right]=\left(\sum_{i=1}^{r}p_{ii}p_{11}+\sum_{i=1}^{r}p_{ii}p_{22}+\cdots +\sum_{i=1}^{r}p_{ii}p_{rr}\right)={\left(\sum_{i=1}^{r}p_{ii}\right)}^{2}.$$

Thus,

$${\left[\frac{\partial \left(miF_{1}\right)}{\partial (p)}\right]}^{T}\left(\text{diag}(p)-pp^{T}\right)\left[\frac{\partial \left(miF_{1}\right)}{\partial (p)}\right]=\left(\sum_{i=1}^{r}p_{ii}\right)\left(1-\sum_{i=1}^{r}p_{ii}\right).$$

Finally,

$$\text{Var}\left(\hat{miF_{1}}\right)=\left(\sum_{i=1}^{r}p_{ii}\right)\left(1-\sum_{i=1}^{r}p_{ii}\right)/n.$$

And

$$\hat{miF_{1}}\dot{\sim }\text{Normal}\left(miF_{1},\left(\sum_{i=1}^{r}p_{ii}\right)\left(1-\sum_{i=1}^{r}p_{ii}\right)/n\right).$$

## Appendix B: Derivation of the distribution and variance of $\hat{maF_{1}}$

In a similar manner to Appendix A, using the multivariate delta-method, we get

$$\sqrt{n}\left(\hat{maF_{1}}-maF_{1}\right)\dot{\sim }\text{Normal}\left(0,{\left[\frac{\partial \left(maF_{1}\right)}{\partial (p)}\right]}^{T}\times \left(\text{diag}(p)-pp^{T}\right)\left[\frac{\partial \left(maF_{1}\right)}{\partial (p)}\right]\right).$$

Now we take the partial derivatives of (2) to get

$$\frac{\partial \left(maF_{1}\right)}{\partial \left(p_{ii}\right)}=\frac{2}{r}\left(\frac{p_{i\cdot }+p_{\cdot i}-2p_{ii}}{{\left(p_{i\cdot }+p_{\cdot i}\right)}^{2}}\right),\;\forall i=1,\cdots r,$$

$$\frac{\partial \left(maF_{1}\right)}{\partial \left(p_{ij}\right)}=\frac{2}{r}\left(\frac{-p_{ii}}{{\left(p_{i\cdot }+p_{\cdot i}\right)}^{2}}+\frac{-p_{jj}}{{\left(p_{j\cdot }+p_{\cdot j}\right)}^{2}}\right),$$

i,j=1,⋯,r;i≠j.

Arranging these terms according to the order of the elements in ***p***, we have

$$\frac{\partial \left(maF_{1}\right)}{\partial (p)}=\frac{2}{r}{\left(\frac{p_{1\cdot }+p_{\cdot 1}-2p_{11}}{{\left(p_{1\cdot }+p_{\cdot 1}\right)}^{2}},\frac{-p_{11}}{{\left(p_{1\cdot }+p_{\cdot 1}\right)}^{2}}+\frac{-p_{22}}{{\left(p_{2\cdot }+p_{\cdot 2}\right)}^{2}},\cdots ,\frac{p_{r\cdot }+p_{\cdot r}-2p_{rr}}{{\left(p_{r\cdot }+p_{\cdot r}\right)}^{2}}\right)}^{T}.$$

Next, we note

$${\left[\frac{\partial \left(maF_{1}\right)}{\partial (p)}\right]}^{T}\left(pp^{T}\right)\left[\frac{\partial \left(maF_{1}\right)}{\partial (p)}\right]=0$$

because

$${\left[\frac{\partial \left(maF_{1}\right)}{\partial (p)}\right]}^{T}p=\frac{2}{r}\left\{\sum_{i=1}^{r}\left(\frac{p_{i\cdot }+p_{\cdot i}-2p_{ii}}{{\left(p_{i\cdot }+p_{\cdot i}\right)}^{2}}\right)p_{ii}-\sum_{i=1}^{r}\left(\frac{\sum_{j\neq i}p_{ij}}{{\left(p_{i\cdot }+p_{\cdot i}\right)}^{2}}\right)p_{ii}-\sum_{j=1}^{r}\left(\frac{\sum_{i\neq j}p_{ij}}{{\left(p_{j\cdot }+p_{\cdot j}\right)}^{2}}\right)p_{jj}\right\}=\frac{2}{r}\left\{\sum_{i=1}^{r}\left(\frac{p_{i\cdot }+p_{\cdot i}-2p_{ii}}{{\left(p_{i\cdot }+p_{\cdot i}\right)}^{2}}\right)p_{ii}-\sum_{i=1}^{r}\left(\frac{\sum_{j\neq i}\left(p_{ij}+p_{ji}\right)}{{\left(p_{i\cdot }+p_{\cdot i}\right)}^{2}}\right)p_{ii}\right\}=0.$$

Therefore,

$${\left[\frac{\partial \left(maF_{1}\right)}{\partial (p)}\right]}^{T}\left(\text{diag}(p)-pp^{T}\right)\left[\frac{\partial \left(maF_{1}\right)}{\partial (p)}\right]={\left[\frac{\partial \left(maF_{1}\right)}{\partial (p)}\right]}^{T}(\text{diag}(p))\left[\frac{\partial \left(maF_{1}\right)}{\partial (p)}\right],$$

which can be shown to equal

$$=\frac{2}{r^{2}}\left\{\sum_{i=1}^{r}\frac{maF_{1i}(p_{i\cdot }+p_{\cdot i}-2p_{ii})}{{\left(p_{i\cdot }+p_{\cdot i}\right)}^{2}}\left(\frac{p_{i\cdot }+p_{\cdot i}-2p_{ii}}{p_{i\cdot }+p_{\cdot i}}+\frac{maF_{1i}}{2}\right)+\sum_{i=1}^{r}\sum_{j\neq i}\frac{p_{ij}maF_{1i}maF_{1j}}{\left(p_{i\cdot }+p_{\cdot i}\right)\left(p_{j\cdot }+p_{\cdot j}\right)}\right\}.$$

Putting all together, we have

$$\hat{maF_{1}}\dot{\sim }\text{Normal}\left(maF_{1},\text{Var}\left(\hat{maF_{1}}\right)\right),$$

where

$$\text{Var}\left(\hat{maF_{1}}\right)=\frac{2}{r^{2}}\left\{\sum_{i=1}^{r}\frac{maF_{1i}\left(p_{i\cdot }+p_{\cdot i}-2p_{ii}\right)}{{\left(p_{i\cdot }+p_{\cdot i}\right)}^{2}}\left(\frac{p_{i\cdot }+p_{\cdot i}-2p_{ii}}{p_{i\cdot }+p_{\cdot i}}+\frac{maF_{1i}}{2}\right)+\sum_{i=1}^{r}\sum_{j\neq i}\frac{p_{ij}maF_{1i}maF_{1j}}{\left(p_{i\cdot }+p_{.i}\right)\left(p_{j\cdot }+p_{\cdot j}\right)}\right\}/n.$$

## Appendix C: Derivation of the distribution and variance of $\hat{maF_{1}^{*}}$

For marcro-averaged precision (*maP*) and macro-averaged recall (*maR*), let the vector ***m*** and its MLE $\hat{m}$ be

$$m=\left(\begin{array}{l} maP\\ maR \end{array}\right),\;\hat{m}=\left(\begin{array}{l} \hat{maP}\\ \hat{maR} \end{array}\right),$$

respectively. Using the multivariate delta-method, we have

$$\sqrt{n}(\hat{m}-m)\dot{\sim }\text{Normal}\left(0_{2},\Sigma \right),$$

where

$$\Sigma =\left[\frac{\partial (m)}{\partial \left(p^{T}\right)}\right]\left(\text{diag}(p)-pp^{T}\right){\left[\frac{\partial (m)}{\partial \left(p^{T}\right)}\right]}^{T}=\left(\begin{array}{l} \left[\frac{\partial (maP)}{\partial \left(p^{T}\right)}\right]\left(\text{diag}(p)-pp^{T}\right){\left[\frac{\partial (maP)}{\partial \left(p^{T}\right)}\right]}^{T},\;\left[\frac{\partial (maP)}{\partial \left(p^{T}\right)}\right]\left(\text{diag}(p)-pp^{T}\right){\left[\frac{\partial (maR)}{\partial \left(p^{T}\right)}\right]}^{T}\\ \left[\frac{\partial (maP)}{\partial \left(p^{T}\right)}\right]\left(\text{diag}(p)-pp^{T}\right){\left[\frac{\partial (maR)}{\partial \left(p^{T}\right)}\right]}^{T},\;\left[\frac{\partial (maR)}{\partial \left(p^{T}\right)}\right]\left(\text{diag}(p)-pp^{T}\right){\left[\frac{\partial (maR)}{\partial \left(p^{T}\right)}\right]}^{T} \end{array}\right)=n\left(\begin{array}{cc} \text{Var}(\hat{maP}) & \text{Cov}(\hat{maP},\hat{maR})\\ \text{Cov}(\hat{maP},\hat{maR}) & \text{Var}(\hat{maR}) \end{array}\right).$$

This is a 2 × 2 matrix with

$$\text{Var}(\hat{maP})=\frac{1}{r^{2}}\left(\sum_{i=1}^{r}\frac{p_{ii}\left(\sum_{j\neq i}p_{ij}\right)}{p_{i\cdot }^{3}}\right)/n,$$

$$\text{Var}(\hat{maR})=\frac{1}{r^{2}}\left(\sum_{i=1}^{r}\frac{p_{ii}\left(\sum_{j\neq i}p_{ji}\right)}{p_{\cdot i}^{3}}\right)/n,$$

$$\text{Cov}(\hat{maP},\hat{maR})=\frac{1}{r^{2}}\left\{\sum_{i=1}^{r}\frac{\left(\sum_{j\neq i}p_{ij}\right)p_{ii}\left(\sum_{j\neq i}p_{ji}\right)}{p_{i\cdot }^{2}p_{\cdot i}^{2}}+\left(\sum_{i=1}^{r}\sum_{j\neq i}\frac{p_{ii}p_{ij}p_{jj}}{p_{i\cdot }^{2}p_{j\cdot }^{2}}\right)\right\}/n.$$

Using the multivariate delta-method again, we get

$$\sqrt{n}\left(\hat{maF_{1}^{*}}-maF_{1}^{*}\right)\dot{\sim }\text{Normal}\left(0,{\left[\frac{\partial \left(maF_{1}^{*}\right)}{\partial (m)}\right]}^{T}\Sigma \left[\frac{\partial \left(maF_{1}^{*}\right)}{\partial (m)}\right]\right),$$

where

$$\frac{\partial \left(maF_{1}^{*}\right)}{\partial (m)}=\left(\begin{array}{l} \frac{2maR^{2}}{{\left(maP+maR\right)}^{2}}\\ \frac{2maP^{2}}{{\left(maP+maR\right)}^{2}} \end{array}\right).$$

Using this and ***Σ*** above, we obtain

$${\left[\frac{\partial \left(maF_{1}^{*}\right)}{\partial (m)}\right]}^{T}\Sigma \left[\frac{\partial \left(maF_{1}^{*}\right)}{\partial (m)}\right]=4n\frac{maR^{4}Var(\hat{maP})+2maP^{2}maR^{2}\text{Cov}(\hat{maP},\hat{maR})+maP^{4}Var(\hat{maR})}{{\left(maP+maR\right)}^{4}}.$$

Finally, we have

$$\hat{maF_{1}^{*}}\dot{\sim }\text{Normal}\left(maF_{1}^{*},\text{Var}\left(\hat{maF_{1}^{*}}\right)\right),$$

where

$$\text{Var}\left(\hat{maF_{1}^{*}}\right)=4n\frac{maR^{4}Var(\hat{maP})+2maP^{2}maR^{2}\text{Cov}(\hat{maP},\hat{maR})+maP^{4}Var(\hat{maR})}{{\left(maP+maR\right)}^{4}}/n.$$

## Appendix D: R code

The following R code computes point estimates and confidence intervals for *miF*_1_, *maF*_1_, and $maF_{1}^{*}$.

## Takahashi et al. ##
## Computation of F1 score and its confidence interval ##

f1scores <- function(mat, conf.level=0.95){
   ## This function computes point estimates and (conf.level*100%) confidence intervals
   ## for microF1, macroF1, and macroF1* scores.

   ## mat is an r by r matrix (confusion matrix).
   ## Rows indicate the predicted (fitted) conditions,
   ## and columns indicate the truth.
   ## miF1 is micro F1
   ## maF1 is macro F1
   ## maF2 is macro F1* (Sokolova and Lapalme)

   ## ###### ##
   ## Set up ##
   ## ###### ##
   r <- ncol(mat)
   n <- sum(mat) ## Total sample size
   p <- mat/n ## probabilities
      pii <- diag(p)
      pi. <- rowSums(p)
      p.i <- colSums(p)

   ## ############### ##
   ## Point estimates ##
   ## ############### ##
      miP <- miR <- sum(pii) ## MICRO precision, recall
   miF1 <- miP ## MICRO F1
      F1i <- 2*pii/(pi.+p.i)
   maF1 <- sum(F1i)/r ## MACRO F1
      maP <- sum(pii/rowSums(p))/r ## MACRO precision
      maR <- sum(pii/colSums(p))/r ## MACRO recall
   maF2 <- 2*(maP*maR)/(maP+maR) ## MACRO F1*

   ## ################## ##
   ## Variance estimates ##
   ## ################## ##

   ## ----------------- ##
   ## MICRO F1 Variance ##
   ## ----------------- ##
   miF1.v <- sum(pii)*(1-sum(pii))/n
   miF1.s <- sqrt(miF1.v)

   ## ----------------- ##
   ## MACRO F1 Variance ##
   ## ----------------- ##

   for(i in 1:r){
         jj <- (1:r)[−i]
      for(j in jj){
         b <- b+ p[i,j]*F1i[i]*F1i[j]/((pi.[i]+p.i[i])*(pi.[j]+p.i[j]))
   }}
   maF1.v <- 2*(a+b)/(n*rˆ2)
   maF1.s <- sqrt(maF1.v)

   ## ------------------ ##
   ## MACRO F1* Variance ##
   ## ------------------ ##

   varmap <- sum(pii*(pi.−pii)/pi.ˆ3) / rˆ2 / n
   varmar <- sum(pii*(p.i−pii)/p.iˆ3) / rˆ2 / n
   covmpr1 <- sum( ((pi.−pii) * pii * (p.i−pii)) / (pi.ˆ2 * p.iˆ2) )
   covmpr2 <- 0
      for(i in 1:r){
         covmpr2 <- covmpr2 + sum(pii[i] * p[i,−i] * pii[−i] / pi.[i]ˆ2 / p.i[−i]ˆ2)
      }
   covmpr <- (covmpr1+covmpr2) / rˆ2 / n
   maF2.v <- 4 * (maRˆ4*varmap + 2*maPˆ2*maRˆ2*covmpr + maPˆ4*varmar) / (maP+maR)ˆ4
   maF2.s <- sqrt(maF2.v)

   ## #################### ##
   ## Confidence intervals ##
   ## #################### ##
   z <- qnorm(1-(1-conf.level)/2)
      miF1.ci <- miF1 + c(−1,1)*z*miF1.s
      maF1.ci <- maF1 + c(−1,1)*z*maF1.s
      maF2.ci <- maF2 + c(−1,1)*z*maF2.s

   ## ################# ##
   ##Formattnig output ##
   ## ################# ##
   pr <- data.frame(microPrecision=miP, microRecall=miR, macroPrecision=maP, macroRecall=maR)
   fss <- data.frame(
         rbind(miF1=c(miF1, miF1.s, miF1.ci),
            maF1=c(maF1, maF1.s, maF1.ci),
            maF1.star=c(maF2, maF2.s, maF2.ci)))
      names(fss) <- c(’PointEst’,’Sd’, ’Lower’,’Upper’)
      out <- list(pr, fss)
      names(out) <- c(’Precision.and.Recall’, ’Confidence.Interval’)
      out
}

## Example ##
## Table V from Dong et al. (2017) PMID: 28767373
mnn <- cbind(c(5022,577,188,19,395),
         c(407,2468,989,4,965),
         c(130,630,27254,1021,763),
         c(13,0,1236,6399,5),
         c(103,258,609,0,9611)
         )

f1scores(mnn)

## End ##
